# The Effectiveness of an App (Insulia) in Recommending Basal Insulin Doses for French Patients With Type 2 Diabetes Mellitus: Longitudinal Observational Study

**DOI:** 10.2196/44277

**Published:** 2023-03-01

**Authors:** Camille Nevoret, Nathalie Gervaise, Brigitte Delemer, Said Bekka, Bruno Detournay, Amine Benkhelil, Amar Bahloul, Geneviève d'Orsay, Alfred Penfornis

**Affiliations:** 1 Biostatistics Department CEMKA Bourg-la-Reine France; 2 Diabetology Department Clinique NCT+ Tours France; 3 Endocrinology, Diabetology and Nutrition Department Robert Debre University Hospital Reims France; 4 Institut de Diabétologie et Nutrition du Centre Mainvilliers France; 5 Diabetes Department Sanofi France Gentilly France; 6 Voluntis SA Suresnes France; 7 Endocrinology, Diabetology and Metabolic Diseases Department Sud-Francilien Hospital Université Paris-Saclay Corbeil-Essonnes France

**Keywords:** diabetes mellitus, insulin, medical informatics apps, telemedicine, mobile health, mobile health intervention, health app, digital monitoring, remote monitoring, virtual care, clinical algorithm

## Abstract

**Background:**

For patients with type 2 diabetes (T2D), calculating the daily dose of basal insulin may be challenging. Insulia is a digital remote monitoring solution that uses clinical algorithms to recommend basal insulin doses. A predecessor device was evaluated in the TeleDiab-2 randomized controlled trial, showing that a higher percentage of patients using the app achieved their target fasting blood glucose (FBG) level compared to the control group, and insulin doses were adjusted to higher levels without hypoglycemia.

**Objective:**

This study aims to analyze how the glycemic control of Insulia users has evolved when using the app in a real-life setting in France.

**Methods:**

A retrospective observational analysis of data collected through the device in adult French patients with T2D treated with basal insulin and oral antihyperglycemic agents using the system for ≥6 months was conducted. Analyses were descriptive and distinguished the results in a subpopulation of regular and compliant users of the app. Glycemic outcomes were estimated considering the percentage of patients who achieved their individualized FBG target between 5.5 and 6 months following the initiation of device use, the frequency of hypoglycemia resulting in a treatment change over the 6-month period of exposure, and the evolution of the average hemoglobin A_1c_ (HbA_1c_) level over the same period.

**Results:**

Of the 484 users, 373 (77.1%) performed at least one dose calculation. A total of 221 (59.2%) users were men. When app use started, the mean age, BMI, HbA_1c_, and basal insulin dose were 55.8 (SD 11.9) years, 30.6 (SD 5.9) kg/m^2^, 10.1% (SD 2.0%), and 25.5 (SD 15.8) IU/day, respectively. Over a median use duration of 5.0 (95% CI 3.8-5.7) months, patients used the system 5.8 (SD 1.6) times per week on average, and 73.4% of their injected doses were consistent with the app’s suggested doses. Among regular and compliant user patients (n=91, ≥5 measurements/week and ≥80% adherence to calculated doses), 60% (55/91) achieved the FBG target (±5%) at 6 months (5.5-6 months) versus 51.5% (145/282) of the other patients (*P*=.15). There was an increase in the proportion of patients achieving their target FBG for regular and compliant users (+1.86% every 2 weeks) without clear improvement in other patients. A logistic model did not identify the variables that were significantly associated with this outcome among regular and compliant users. In the overall population, the incidence of reported hypoglycemia decreased simultaneously (–0.16%/month). Among 82 patients, the mean HbA_1c_ decreased from 9.9% to 7.2% at 6 months.

**Conclusions:**

An improvement in glycemic control as measured by the percentage of patients reaching their FBG individualized target range without increasing hypoglycemic risk was observed in patients using the Insulia app, especially among regular users following the dose recommendations of the algorithm.

## Introduction

For patients with type 2 diabetes (T2D), achieving recommended glycemic targets remains difficult, especially in people treated with basal insulin. One of the reasons for this difficulty is related to the challenge of titrating insulin doses. Insulia is a digital solution combining a smartphone app for basal insulin dose suggestions and a web portal accessible to professionals to personalize and manage patients’ treatments remotely. Beyond remote monitoring of basal insulin therapy, the app uses the data entered by the patients to calculate the recommended basal insulin dose according to the objectives set by the patient’s physician. This dose calculation is triggered by the patient’s request.

A predecessor device to Insulia called Diabeo-Basal was evaluated in the TeleDiab-2 study [1]. This randomized controlled trial evaluated the efficacy and safety of two remote monitoring systems to optimize basal insulin initiation in patients with poorly controlled T2D (hemoglobin A_1c_ [HbA_1c_] 7.5%-10%). A total of 191 participants (mean age 58.7 years, mean HbA_1c_ 8.9%) were randomized into three groups: group 1 (standard care, n=63), group 2 (interactive voice response system, n=64), and group 3 (Insulia app software, n=64). After 4 months of follow-up, HbA_1c_ reduction was significantly higher in the remote monitoring groups (group 2: –1.44% and group 3: –1.48% vs group 1: –0.92%; *P*=.002). In addition, twice as many patients in the telemonitoring groups achieved their target fasting blood glucose (FBG) level as in the control group, and insulin doses were adjusted to higher levels. No severe hypoglycemia was observed in the remote monitoring groups, and the frequency of mild hypoglycemia was similar in all groups.

Consequently, Insulia was available by prescription and used in France as part of a nationwide program financing new health remote monitoring systems (Expérimentations de télémédecine pour l'amélioration des parcours en santé [ETAPES] program: National Experiments on Remote Diabetes Monitoring [2]) since the end of 2020. Despite the potential benefits for patients suggested by the TeleDiab-2 study, Insulia, as with other apps offering insulin dose calculation, carries a risk of incorrect dose recommendations, which could lead to suboptimal disease control [3]. In this field, perhaps even more than for other health products, it is necessary to complement experimental results with the analysis of monitoring data on both the clinical efficacy and safety of the apps during their use in real life.

The data entered by the patients and their physicians in the Insulia app are collected on a dedicated computer platform. This study aims to analyze this database to determine how the glycemic control of Insulia users has evolved when using the app in a real-life setting in France.

## Methods

### Overview

The Insulia app is presented in Multimedia Appendix 1. From the Insulia app’s home screen, patients can enter their blood glucose monitoring, hypoglycemia symptoms, and insulin doses. Insulia takes this data into account to recommend personalized doses in real time. Each recommended dose is accompanied by an explanation of how it was calculated. Data is automatically sent to the health care team so that they can monitor the patient’s progress and even adjust the treatment. The ETAPES program funds the device for 6 months for patients with T2D diagnosed more than 12 months ago, who are 18 years or older, with an HbA_1c_ ≥9% on two measurements taken within a 6-month interval, and treated with insulin. It also funds the device for a maximum of 3 months in patients with T2D diagnosed for more than 12 months who were 18 years or older at the time of insulin initiation when their HbA_1c_ level was <9% at two measurements taken within a 6-month interval.

A retrospective observational study was conducted using data collected through the Insulia device in adult patients with T2D who were treated with basal insulin and oral antihyperglycemic regimen and who were enrolled as users of the solution for 6 months or more by September 30, 2021.

A subpopulation of regular and compliant users was identified. These are patients who have used the device for at least 6 months without interruption with at least 5 dose calculations per week on average during the study period and for whom more than 80% of their injected insulin doses corresponded to the recommended doses.

Glycemic outcomes were estimated considering the percentage of patients who achieved their individualized glycemic target (average FBG level ±5%) between 5.5 and 6 months following the initiation of the device use.

Other criteria included the frequency of hypoglycemia resulting in a change in treatment over the 6-month period of exposure and the evolution of the average HbA_1c_ level over the same period of time (+/– 1.5 months). HbA_1c_ level was not considered as the primary glycemic outcome as the collection of this data is not mandatory in the app, which determines the insulin doses to be administered based on FBG levels.

A multivariate regression analysis was finally conducted on the achievement of the FBG objective, including a subgroup analysis considering the patients from the center with the most patients versus other patients to identify a possible center effect.

### Ethical Considerations

This study was conducted in accordance with Regulation (EU) 2016/679 of the European Parliament and of the Council of April 27, 2016, on the protection of natural persons with regard to the processing of personal data and the free movement of such data. Informed consent of the patients was not requested as the data analyzed were fully anonymized. A full privacy impact assessment was conducted on July 29, 2021.

## Results

The Insulia database included 484 patients enrolled as users of the app for 6 months or more. Among them, 111 patients did not conduct any dose calculation with the device or did not indicate any basal insulin dose injected since their registration on the app. Consequently, 373 patients were considered in the main analysis. Among them, 91 (24.4%) patients were identified as regular and compliant users over a 6-month period.

The characteristics of the patients are described in Table 1. On average, they were aged 55.8 (SD 11.9) years, and 59.2% (n=221) were men. At the time of their first use of the Insulia device, 48.6% (n=181) of them had a BMI ≥30 kg/m^2^ (average BMI 30.6 kg/m^2^). The mean HbA_1c_ level was 10.1% (SD 2%). The individual FBG target ranged from 70-100 to 100-150 mg/dL. The target ranges were 80-130 mg/dL for 33.5% (n=125) of the patients and 80-120 mg/dL for 30% (n=112) of the patients. The first calculated basal insulin dose averaged 25.5 IU with significant variability (between 4 IU and 92 IU according to the patients). Among compliant and regular users (n=91, 24.4% of the patients over the 6 months of observation), the HbA_1c_ level at baseline was slightly lower compared to other patients (9.6% vs 10.3%; *P*=.002), and the FBG target was slightly more stringent, with a higher proportion of patients having an FBG target in the range of 80-120 mg/dL and a lower proportion in the range of 100-150 mg/dL.

**Figure 1:**
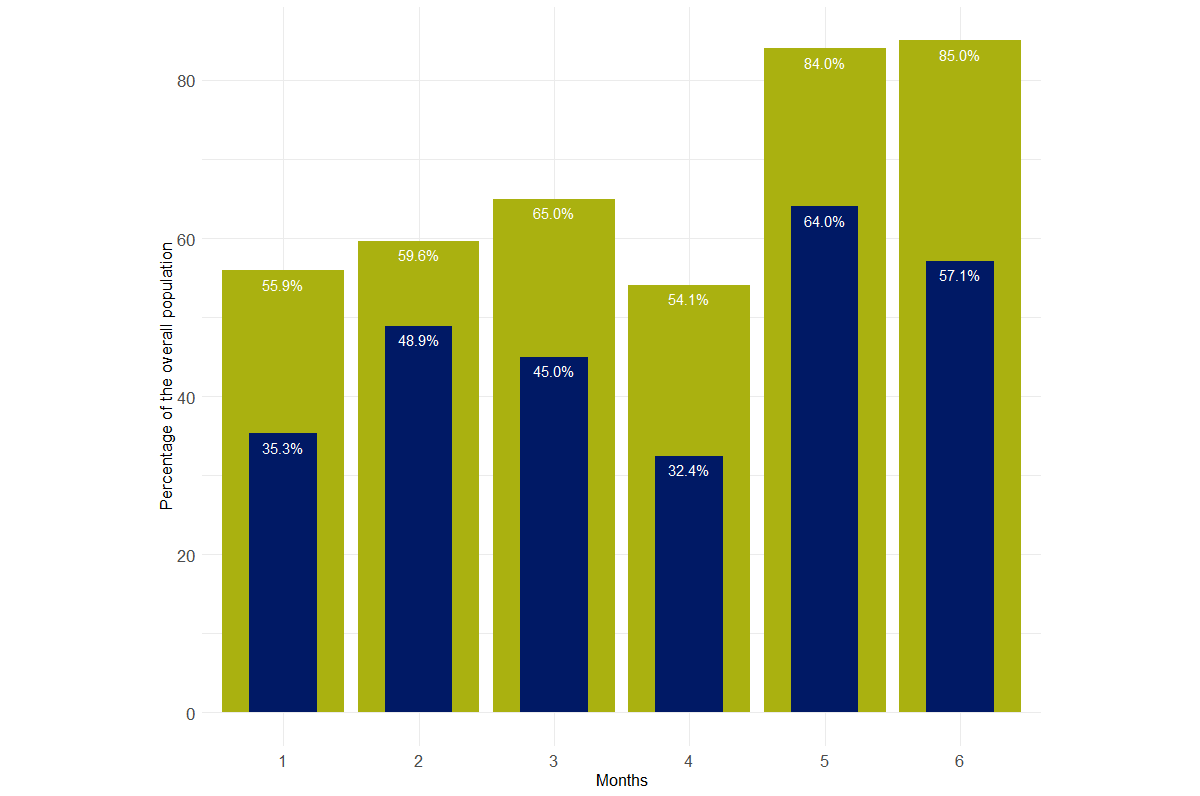
Percentage of patients who were regular and compliant Insulia users over time.

The percentage of patients defined as regular and compliant users evolved during the 6-month period of the study with a progressive disaffection of the patients from the second month of use. A similar evolution was observed considering only regular use of the device (at least 5 dose calculations per week; Figure 1).

**Figure 2:**
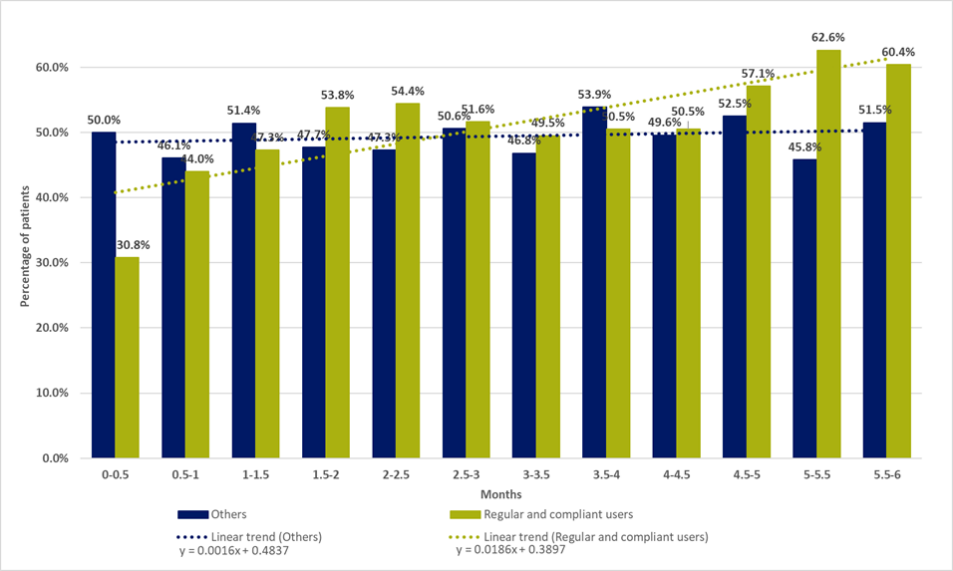
Percentage of Insulia users achieving their individualized fasting blood glucose target over time.

Figure 2 shows the percentage of Insulia users with an average FBG in their individualized target range over 15-day periods according to whether they are regular and compliant users. The percentages are calculated on the number of patients still using the device over the 15-day period. We observed an increase in the proportion of patients achieving their target FBG for regular and compliant users (+1.86% every 2 weeks). No clear improvement was observed in other patients (irregular or not compliant users of the app).

After 6 months of use (5.5 to 6 months), the FBG target was achieved in 60% (55/91) of the regular and compliant users versus 51.5% (145/282) of the other patients (*P*=.15), although the FBG target was slightly more stringent for the regular and compliant users.

**Figure 3:**
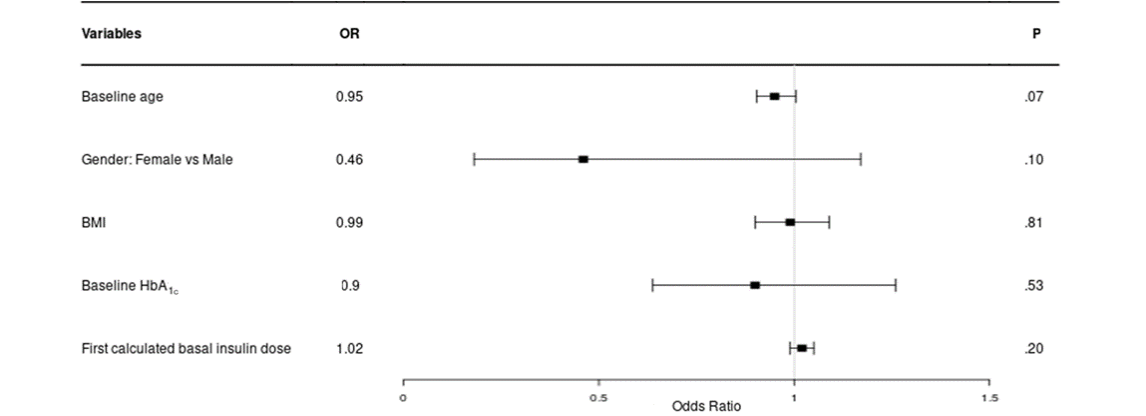
Explanatory logistic model for achieving the individualized fasting blood glucose target after 6 months among regular and compliant users (n=91). HbA_1c_: hemoglobin A_1c_; OR: odds ratio.

Variables available at baseline (age, gender, BMI, HbA_1c_ level, and insulin dose at Insulia initiation) were tested in a logistic model to explain potential factors associated with achieving the individualized FBG target at 6 months among regular and compliant users (n=91; Figure 3). None of these variables were significantly associated with this outcome.

**Figure 4:**
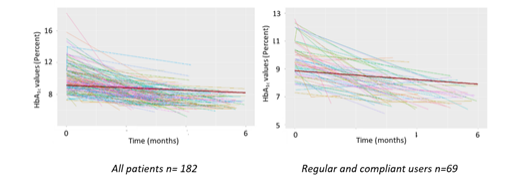
Trends in HbA_1c_ level evolution over 6 months of Insulia app use. HbA_1c_: hemoglobin A_1c_.

Figure 4 presents the evolution of the HbA_1c_ level over time for patients having at least 2 measurements regardless of the time elapsed between these two measurements (all patients, n=182, and all regular and compliant users, n=69). In both cases, a slight but significant decrease in HbA_1c_ values of –0.155% and –0.161% per month, respectively, was observed.

Data were available at baseline and 6 months for only 82 patients. The mean HbA_1c_ level decreased from 9.9% to 7.2% after 6 months (±1.5 months) of app use, with no significant difference according to the degree of Insulia use.

Table 2 shows the numbers and proportions of patients who reported in the app that they had at least one change in treatment because of hypoglycemia, defined as a blood glucose measurement <70 mg/dL and whether this hypoglycemia was symptomatic or not. A favorable trend (–0.16% per month) was observed but not statistically significant due to the low number of patients.

Finally, to identify a possible center effect, a subgroup analysis was conducted on the sample of patients enrolled in the principal investigating center (Centre Hospitalier Sud Francilien [CHSF], Corbeil-Essonnes) where 68 (18.3%) patients of the overall population considered in the analysis (N=373) were enrolled. The baseline characteristics of those patients were similar to those of other centers, excluding a higher average HbA_1c_ level at the time of the first basal insulin dose calculation (10.9%, SD 2.3% vs 10.0%, SD 1.9%). Interestingly, individualized FBG targets were also often less strict in this center than in other centers (patients with a target between 100-150 mg/dL: 20/68, 29.4% vs 12/305, 3.9%), and over the first 6 months, patients at the CHSF were less often regular and compliant users than at the other centers (9/68, 13.2% vs 82/305, 26.9%; *P*=.02). Despite these discrepancies, the percentage of Insulia app users achieving their FBG target after 6 months was not different considering the overall population (*P*=.77) or only regular and compliant users (*P*=.75), excluding a center effect on the results.

**Table 1:** Patients’ characteristics (at the time of the first basal insulin dose calculation).

			Regular and compliant users (n=91)	Other patients (n=282)	Total (N=373)	*P* value		
Gender (male), n (%)			52 (57.1)	169 (59.9)	221 (59.2)	.64		
Age (years), mean (SD)			58.0 (9.4)	55.3 (12.2)	55.8 (11.9)	.06		
**Age (years), n (%)**								.10
	<40		1 (1.1)	29 (10.3)	30 (8.3)			
	40-50		17 (18.7)	54 (19.2)	71 (19.0)			
	50-60		35 (38.5)	90 (31.9)	125 (33.4)			
	60-70		28 (30.8)	77 (27.3)	105 (28.1)			
	70-80		8 (8.8)	29 (10.3)	37 (10.0)			
	≥80		2 (2.2)	3 (1.1)	5 (1.3)			
**Clinical characteristics**								
	BMI (kg/m^2^), mean (SD)		31.0 (5.0)	30.4 (6.1)	30.6 (5.9)	.49		
	**BMI (kg/m^2^), n (%)**						.06	
		<26.5	15 (16.5)	77 (27.3)	92 (24.7)			
		26.5-30	33 (36.6)	67 (23.8)	100 (26.8)			
		30-35	25 (27.5)	80 (28.4)	105 (28.2)			
		≥35	18 (19.8)	58 (20.6)	76 (20.4)			
	**HbA_1c_^a^ level (%)**						.002	
		Mean (SD)	9.6 (1.4)	10.3 (2.1)	10.1 (2.0)			
		Median (min, max)	9.5 (6.8, 13.0)	9.8 (6.0, 18.8)	9.7 (6.0, 18.8)			
		Quartile 1, quartile 3	8.7, 10.3	9.0, 11.5	8.8, 11.2			
	**FBG^b^ target as defined by the practitioner (mg/dL)**						.02	
		80-130	30 (33.0)	95 (33.7)	125 (33.5)			
		80-120	36 (39.6)	76 (27.0)	112 (30.0)			
		100-150	2 (2.2)	30 (10.6)	32 (8.6)			
		Other	23 (25.3)	81 (28.7)	104 (27.9)			
**Treatment**								
	**First calculated insulin dose (UI)**						.58	
		Mean (SD)	26.3 (17.1)	25.2 (15.4)	25.5 (15.8)			
		Median (min, max)	22.0 (4.0, 74.0)	20.0 (4.0, 92.0)	20.0 (4.0, 92.0)			
		Quartile 1, quartile 3	14.0, 32.0	14.0, 32.0	14.0, 32.0			

^a^HbA_1c_: hemoglobin A_1c_.

^b^FBG: fasting blood glucose.

**Table 2:** Percentage of patients reporting a hypoglycemic episode over a 6-month period using the Insulia app.

Month of use	Patients with at least one BG^a^ measure <70 mg/dL, n (%)	Patients with at least one BG measure <70 mg/dL with symptomatic hypoglycemia, n (%)	Patients with at least one BG measure <70 mg/dL with asymptomatic hypoglycemia, n (%)
0-1	17 (17.7)	14 (15.4)	5 (5.5)
1-2	15 (16.5)	12 (13.2)	3 (3.3)
2-3	18 (19.8)	16 (17.6)	2 (2.2)
3-4	12 (13.2)	9 (9.9)	5 (5.5)
4-5	12 (13.2)	11 (12.1)	1 (1.1)
5-6	9 (9.9)	9 (9.9)	1 (1.1)

^a^BG: blood glucose.

## Discussion

### Principal Findings

More technologies are being developed to assist in outpatient insulin dosing [4], but few of them are intended to adjust long-acting insulins for patients with T2D. After reviewing patients’ data, medical history, comorbidities, and current treatment, providers formulate initial insulin dose and titration plans. A target blood glucose range is defined individually as other criteria including adjustment period, low blood glucose threshold, and maximum total daily doses. Patients are supposed to log their FBG readings and episodes of hypoglycemia events or administered insulin doses. Based on these inputs, the system recalculates the next appropriate dose of basal insulin. Franc et al [1] reported on the TeleDiab-2 trial that, at month 4, twice as many patients using such a device compared to the control group achieved an HbA_1c_ level <7% (29.8% vs 12.5%). Other similar devices have also shown positive results [5,6]. However, the translation of clinical trial results into real life often raises a series of questions that lead to an interest in conducting postmarketing observational studies of products. This is especially the case when the assessed technology is strongly dependent on the involvement of the patients who use it as well as on the nature of the support implemented by the professionals around the technology.

Following the marketing of the Insulia device in France, we aimed to examine the results obtained in real life by the users of such a solution. This study was conducted based on data collected through the system itself, which constitutes a methodological limitation due to the relatively large number of missing data on some outcomes (ie, HbA_1c_ level evolution). Nevertheless, some results were of interest. First, the device cannot be expected to have a positive effect if it is not used by the patient. About one-fifth of the patients did not use it after the first inscription on the device, and among users, only 37% (138/373) were still regular users, and 24.4% (91/373) were regular and compliant users after 6 months. We noted a progressive disaffection of the patients with time; even in the first 3 months, only half of the patients were regular users and slightly more than one-third of the patients were both regular and compliant users.

As anticipated, the impact on glycemic control was significantly better among regular and compliant users, with an individualized FBG target achieved in 60% (55/91) of patients after 6 months versus 51.5% (145/282) in other patients using Insulia less frequently. The trend in the proportion of patients achieving their target FBG (+1.86% every 2 weeks for regular and compliant users) and the HbA_1c_ level decrease (from 9.9% to 7.2%) after 6 months (±1.5 months) of app use are in the same direction as the results obtained in clinical trials but were clearly less favorable. The patient selection and close monitoring generally implemented in clinical trials is one possible explanation for this situation. Another explanation probably lies in the fact that, in the TeleDiab-2 study as well as the Bergenstal et al [7] study, the included patients benefited from sustained human support, which was not necessarily the case in our real-life observational study. This probably reflects the importance of the support that must be provided to patients when a tool such as Insulia is offered to them. It also highlighted the necessity to conduct as often as possible pragmatic trials to estimate the added value of such devices.

### Conclusion

In real life, an improvement in glycemic control as measured by the percentage of patients reaching their FBG individualized target range without increasing the incidence of hypoglycemia was observed in patients regularly using the Insulia app and following the dose recommendations of the algorithm. However, these results should be confirmed on a larger population as no significant difference according to the degree of Insulia use was observed considering HbA_1c_ level results.

## Appendix

Multimedia Appendix 1 Insulia app description.

## Abbreviations

CHSF: Centre Hospitalier Sud Francilien
ETAPES: Expérimentations de télémédecine pour l’amélioration des parcours en santé
FBG: fasting blood glucose
HbA_1c_: hemoglobin A_1c_
T2D: type 2 diabetes
