# Supplementary material for: The Effectiveness of an App (Insulia) in Recommending Basal Insulin Doses for French Patients With Type 2 Diabetes Mellitus: Longitudinal Observational Study
Source: JMIR Diabetes. 2023 Mar 1;8:e44277. doi: 10.2196/44277 (PMC10018375; doi:10.2196/44277)

**Insulia® app description**

‘Insulia’ is a prescription-only, software medical device developed by Voluntis Inc., that provides basal insulin dose recommendations and educational coaching messages based on blood glucose values and health events.

Insulia is intended for patients meeting the following criteria:

- Adults with type 2 diabetes
- Treated with basal Insulin (Basal only or Basal bolus)
- Not pregnant
- Not using an insulin pump or pre-mix insulin
- Smartphone, tablet or PC/internet user

The healthcare professionals managing the patient can set an account and treatment plan via a web portal. Information required includes:

- Patient personal and contact details
- Current measures:
  - Weight
  - Height
  - Last HbA1c level
- Blood glucose level goals
- Insulin prescription
  - Brand of insulin
  - Number of injections each day
  - Starting dose
- Adjustment settings:
  - Time period for dose adjustments
  - Units of does increase (minimum and maximum)
  - Maximum daily dose

Once the healthcare professional has set the patient up on the web-portal they then receive an email with details of how to set up and start using the app. The patient accesses a mobile app to enter blood glucose readings and any hypoglyceamic events and the recommended insulin dose is then calculated by the app. The patient then confirms the amount of insulin injected by entering this into the app. An image of the module app is given in figure 1.

The data entered by the patient is automatically transferred to a secure cloud environment and can be seen by the healthcare professional on the web portal. The analysis and reporting features of the web application provide treatment overview, charts and graphs for use by the HCP. In addition, Insulia provides the HCP with automated notifications related to the patient’s BG history and use of the medical device, for example if the patient has not recently entered any blood glucose readings, or there has been a hypoglycemic episode. These notifications are only seen when logged onto the web portal. Healthcare professionals can also see an overview of their patients, for example, of those patients actively using the app the percentages with difference HbA1c levels can be seen.

Figure 1 – The Insulia app


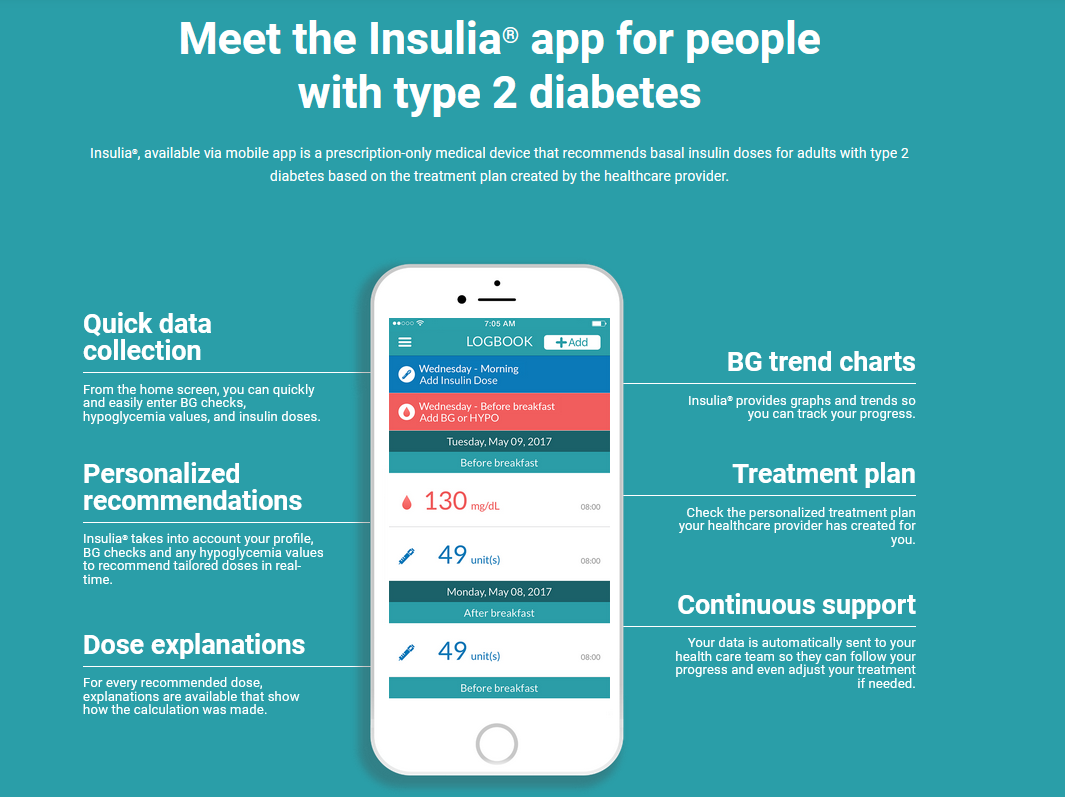

Supplement: Multimedia Appendix 1 [file diabetes_v8i1e44277_app1.docx]
